# Predicting factors for malaria re-introduction: an applied model in an elimination setting to prevent malaria outbreaks

**DOI:** 10.1186/s12936-016-1192-y

**Published:** 2016-03-02

**Authors:** Mansour Ranjbar, Alireza Shoghli, Goodarz Kolifarhood, Seyed Mehdi Tabatabaei, Morteza Amlashi, Mahdi Mohammadi

**Affiliations:** Center for Vectors and Vector-Borne Diseases, Department of Biology, Mahidol University, Bangkok, Thailand; Zanjan Social Determinants of Health Research Centre, Zanjan University of Medical Silences and Health Services, Zanjan, Iran; Epidemiology Department, School of Public Health, Shahid Beheshti University of Medical Silences and Health Services, Tehran, Iran; Infectious Diseases and Tropical Medicine Research Center, School of Public Health, Zahedan University of Medical Sciences, I.R. of Iran, Zahedan, Iran; Independent Malaria Consultant, Tehran, Iran; Health Promotion Research Center, Zahedan University of Medical Sciences, Zahedan, Iran; Independent Malaria Consultant, Member of Surveillance, Monitoring and Evaluation Technical Expert Group, Global Malaria Programme, WHO, Geneva, Switzerland

**Keywords:** Malaria, Re-introduction, Elimination, Outbreak, Forecast, MEWS, Transmission, Meteorological, Population movement

## Abstract

**Background:**

Malaria re-introduction is a challenge in elimination settings. To prevent re-introduction, receptivity, vulnerability, and health system capacity of foci should be monitored using appropriate tools. This study aimed to design an applicable model to monitor predicting factors of re-introduction of malaria in highly prone areas.

**Methods:**

This exploratory, descriptive study was conducted in a pre-elimination setting with a high-risk of malaria transmission re-introduction. By using nominal group technique and literature review, a list of predicting indicators for malaria re-introduction and outbreak was defined. Accordingly, a checklist was developed and completed in the field for foci affected by re-introduction and for cleared-up foci as a control group, for a period of 12 weeks before re-introduction and for the same period in the previous year. Using field data and analytic hierarchical process (AHP), each variable and its sub-categories were weighted, and by calculating geometric means for each sub-category, score of corresponding cells of interaction matrices, lower and upper threshold of different risks strata, including low and mild risk of re-introduction and moderate and high risk of malaria outbreaks, were determined. The developed predictive model was calibrated through resampling with different sets of explanatory variables using R software. Sensitivity and specificity of the model were calculated based on new samples.

**Results:**

Twenty explanatory predictive variables of malaria re-introduction were identified and a predictive model was developed. Unpermitted immigrants from endemic neighbouring countries were determined as a pivotal factor (AHP score: 0.181). Moreover, quality of population movement (0.114), following malaria transmission season (0.088), average daily minimum temperature in the previous 8 weeks (0.062), an outdoor resting shelter for vectors (0.045), and rainfall (0.042) were determined. Positive and negative predictive values of the model were 81.8 and 100 %, respectively.

**Conclusions:**

This study introduced a new, simple, yet reliable model to forecast malaria re-introduction and outbreaks eight weeks in advance in pre-elimination and elimination settings. The model incorporates comprehensive deterministic factors that can easily be measured in the field, thereby facilitating preventive measures.

**Electronic supplementary material:**

The online version of this article (doi:10.1186/s12936-016-1192-y) contains supplementary material, which is available to authorized users.

## Background

In the period 2000–2013, substantial reduction of malaria mortality and morbidity in the world was achieved that resulted in the acceleration of efforts towards malaria elimination [1]. In the Global Technical Strategy for Malaria (2016–2030), the vision of a world free of malaria has been highlighted and at least 35 countries with the continuous transmission in 2015 aim to achieve malaria elimination by 2030 [2]. While the concept of eliminating malaria is bold, there are 100 endemic countries with continuous malaria transmission and the main concern is malaria transmission re-introduction in malaria-free areas worldwide through population movement with endemic countries [3], e.g., in eastern Mediterranean region, re-introduction of malaria has occurred more than once in countries that had been free from malaria [3].

To reduce local cases and to maintain elimination status, the focus should be on monitoring receptivity, vulnerability, and health system capacity [4]. There is enough evidence to support the role of health systems in monitoring malaria disease through early case detection and appropriate response to prevent re-introduction, especially in malaria-prone areas [5–8]. In this regard, to prevent re-introduction of malaria transmission and malaria outbreaks, factors triggering transmission, such as human, vector and parasite factors, should regularly be monitored [9].

Resorting to new tools to monitor susceptibility to malaria occurrence is inevitable [10]. Numerous studies were conducted for prediction of malaria epidemics in endemic countries and the necessity for malaria early warning systems (MEWS) has been emphasized [11, 12]. Accordingly, in malaria control settings, variables such as population movement, minimum and maximum temperatures, rainfall, and humidity were suggested when designing MEWS [13–15]. Each country is exposed to particular and different ecological circumstances; challenges are more apparent at local level when using similar indicators to predict malaria occurrence [16]. A limited number of studies focus on a comprehensive approach for early warning, which considers predictive factors rather than meteorological variables [17]. This is a crucial issue in pre-elimination and elimination settings where variables other than meteorological are more important. However, only a handful of studies have focused on forecasting malaria outbreaks or re-introduction in elimination settings [13, 14, 18].

Iran started its malaria elimination programme in 2009 and has experienced a dramatic decline in the number of malaria cases over the last 6 years. As highlighted in its National Strategic Plan for Malaria Elimination, one of the main concerns to achieve malaria elimination is how to prevent re-introduction malaria transmission. This study aimed to design an applicable model for pre-elimination and elimination settings to assess a comprehensive list of predicting factors for re-introduction in malaria-prone areas and to predict the possibility of malaria outbreaks.

## Methods

### Malaria situation in Iran: study area

A mixed method study was undertaken in a pre-elimination setting in Sistan and Baluchistan Province. The province is populated by around 2.5 million persons, of whom 51 % are rural. According to the human development index (HDI), it is the most underdeveloped region in Iran, with the highest rate of population growth [19]. About 14 % of rural households have no access to electricity [20]. In 2013, around 84 % of foci with local transmission were located in this province, where both *Plasmodium vivax* and *Plasmodium falciparum* have been reported; 10 % were estimated to be *P. falciparum*. Due to the implementation of elimination strategy, the local transmission was limited to less than 3 % of rural foci in Sistan and Baluchestan in 2013, the majority with a population of less than 400. Malaria in this region follows an unstable pattern with two transmission seasons: from March to May, and from July to October, with annual average temperature and relative humidity ranging from 22 to 37 °C and 31 to 76 % min and max, respectively, based on district ground synoptic weather stations reports [21]. The area borders malaria-endemic regions of Afghanistan and Pakistan, where population movement between Iran, Afghanistan and Pakistan is a routine practice, with its consequent risk of re-introduction. Moreover, the presence of marginalized people on low income, with high rate of illiteracy, limited access to air-conditioning systems, the climatic conditions, and the presence of the main vectors, makes this region a high-risk area for malaria transmission.

Since the Malaria Elimination Programme has been undertaken in Iran, everybody has access to free of charge, active and passive case-finding services, the minimum of annual blood examination rate (ABER) in the study areas for the period of 2008–2013 was 18 % [22]. In addition, the early detection system for timely and complete reporting of detected malaria cases had been established for more than a decade. Therefore, missing a re-introduced case seems a very unlikely possibility.

### Variable identification process, checklist design and field study

To develop a checklist of 20 explanatory variables, the nominal group technique was used by a group consisting of epidemiologists, entomologists, parasitologists, clinicians, and health system specialists with at least 5 years’ field experience in malaria areas, together with a literature review of international and national sources (databases of PubMed, ScienceDirect, Scopus, Web of Science, Iranmedex, Sientific Information Database) was conducted. The keywords for the literature review were ‘malaria’ in conjunction with ‘re-introduction’, ‘elimination’, ‘outbreak’, ‘forecast’, ‘MEWS’, ‘transmission’, ‘meteorological variables’, and ‘population movement’. In order to develop a user-friendly model for applicants in the field, three sub-categories for each variable were considered, each indicating its impact severity (a range of high, moderate, low) on triggering re-introduction and malaria outbreaks. The checklist included five main components: (1) parasite variables; (2) history of malaria and other disease outbreaks in the focus; (3) access to the health services; (4) meteorological variables; and, (5) vector variables (see Additional file 1: questionnaire). Next, 33 rural foci affected by re-introduction of malaria transmission were selected once in a six-year period from March 2008 to March 2013. In this study, a focus was considered to be ‘a defined and circumscribed locality situated in a currently or formally malarious area and containing the continuous or intermittent epidemiological factors necessary for malaria transmission’ [23], and re-introduction of malaria transmission was considered to be ‘resurgence of malaria transmission (*P. falciparum* or *P. vivax*, or both) in a cleared-up focus’. Cleared-up foci were defined as ‘foci with no history of malaria transmission within the previous 36 months’. The inclusion criterion for selecting a focus affected by re-introduction was the occurrence of locally transmitted malaria case(s) during three successive weeks.

**Fig. 1:**
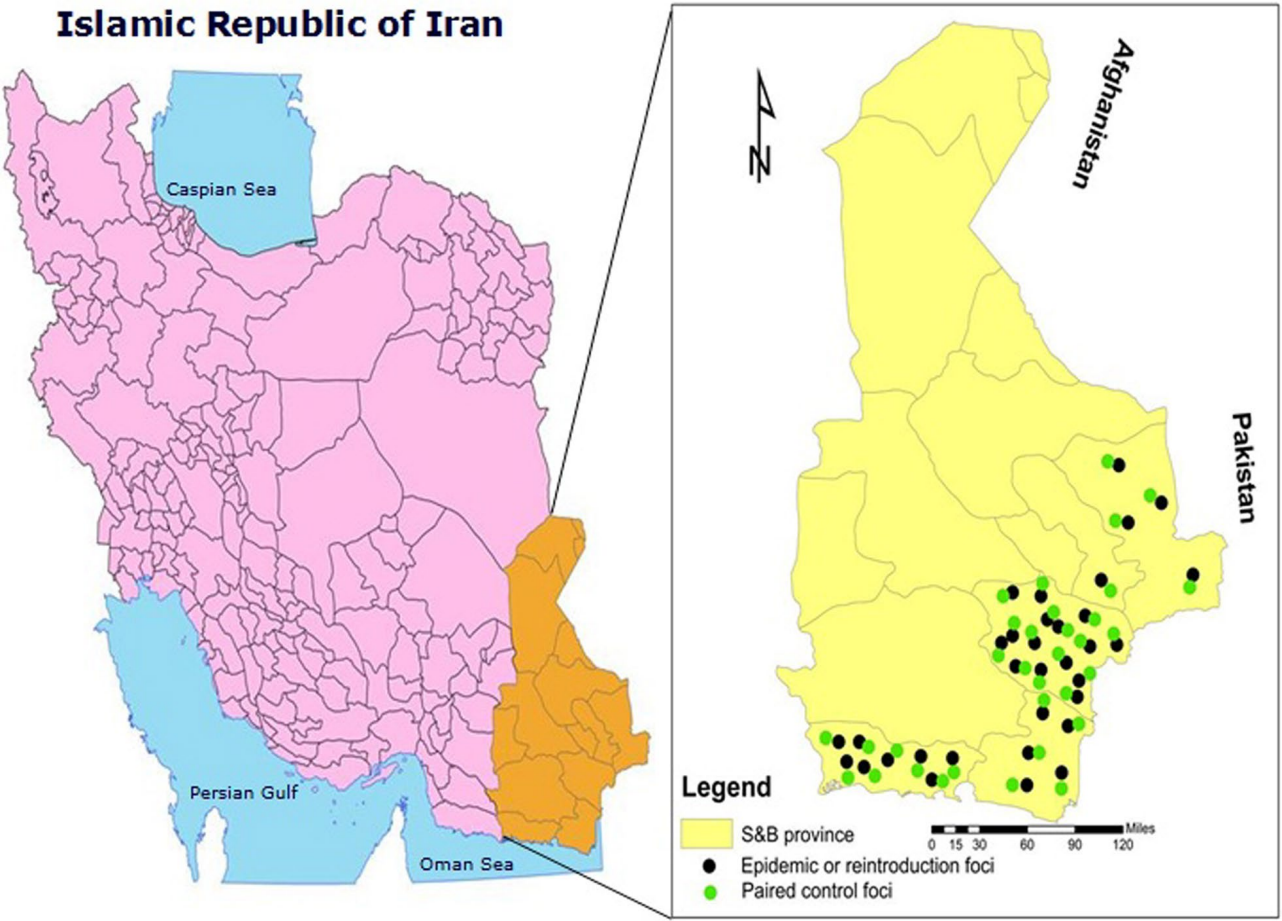
A map of the study area in Sistan and Baluchistan province showing affected and paired control foci

For each selected focus with re-introduction, a cleared-up focus with no history of local malaria cases during the previous 3 years was selected as a paired control focus to find triggering variables for re-introduction and to exclude interfering environmental factors, particularly meteorological variables. These paired control foci were located the closest possible distance from the foci affected by re-introduction and with a similar range of population (Fig. 1). Considering the similarity of geographical and the meteorological condition between focus affected by re-introduction and paired control, the affected focus with re-introduction were compared against its condition at the same period in the previous year when the focus had been classified as cleared-up with no report of local cases.

In the next step, a training event was conducted and the checklist was introduced by the research team to a new group of experienced staff with at least 10 years’ experience in the field of malaria, in addition to their educational background of entomology and epidemiology. The checklist was filled out in the field for an affected focus by re-introduction and also for its paired control focus. The checklist was completed in two different time periods for the focus affected by re-introduction: (1) for a period of 12 weeks prior to diagnosis of the first case after the re-introduction; and, (2) for the same period (for 12 weeks) the previous year with no case reports. In addition, it was completed for the paired control focus for 12 weeks coinciding with the resurgence of malaria transmission in the focus.

### Weighting process for variables

Given data from the field study and collecting the meteorological data from district ground synoptic weather stations, sub-categories of each variable were weighted by an expert group, with numerical values ranging from 0.01 to 10 (weakest to strongest predictors of the epidemic in next 8 weeks, respectively) based on relative frequency difference between the affected and control foci. The sum of sub-category values for each variable was considered to be 9 (100 %). Finally, based on the weights for the sub-categories, 20 selected variables, using pair-wise comparison matrix, were prioritized in view of their impact on triggering re-introduction of malaria transmission by Expert Choice software version 11 suggested by analytic hierarchical process (AHP) [24, 25].

### Mathematical approach

According to AHP output, once the hierarchy was made, the most effective variable (named Ø) with the highest score was defined as a trigger of malaria re-introduction and outbreak. To define critical thresholds of four categories for stratification of the risk of re-introduction as well as an epidemic, 19 L-shaped matrices of 3 × 3 cells were made, the product of sub-categories of Ø on other variables (named Ɵ’s) were determined (see Additional file 2: Product of weights). The matrix was developed based on idea of Haddon matrix, which is used by public health decision-makers, especially in injury prevention settings [26]. Haddon matrix output is a qualitative analysis of emergent conditions which was added to an innovative mathematical approach to analyse and interpret the model output in a quantitative manner. In the second step, the geometric means (GMs) for corresponding cells of 19 matrixes were calculated by sub-category scores of Ɵ and Ø (see Additional file 3: Geometric mean calculation).

Four strata for the risk of malaria re-introduction or epidemics were obtained where:

- GM for the matrix cells of ØL × ƟiL and ØL × ƟiM were considered as lower and upper bounds of ‘low risk of re-introduction’ range, respectively;
- GM for the matrix cells of ØH × ƟiL and ØM × ƟiM were considered as lower and upper bounds of ‘mild risk of re-introduction’ range, respectively;
- GM for the matrix cells of ØM × ƟiM and ØH × ƟiM were considered as lower and upper bounds of ‘moderate risk of outbreak’ range, respectively;
- GM for the matrix cells of ØH × ƟiM and ØH × ƟiH were considered as lower and upper bounds of ‘high risk of outbreak’ range, respectively.

$$\theta_{i} ;i = 1,\ldots,19$$

### Calibration of the model

Using R software version 3.1.3, for any given stratum of Ø, 90 repeated random samples of remaining 19 variables (Ɵ’s) were generated in their similar sub-categories of high, moderate and low. The sampling was done with three different probabilities of 10–20, 21–79 and 80–90 % and their complementary probabilities for Ɵi (e.g., at ØH, ten repeated random samples of ƟiH with probability of 80 % and complementary probability, 20 %, for other variables of ƟiM in a category of moderate risk, were selected). Finally, 270 random samples with different risk sets, the numbers of GM scores with similar results in the range of four risk prediction categories proposed by the model (GMs in the similar range of each defined risk category of re-introduction and outbreaks prediction) were considered as the cut-offs for accuracy.

### The model sensitivity and specificity

To determine the model sensitivity and specificity, a retrospective case–control study was conducted using the National Database of Malaria Foci from 2014 to 2015. Accordingly, 20 foci with re-introduction of malaria were randomly selected from different districts in Iran. In addition, data of all seven foci with malaria outbreaks in 2014 and 2015 (with four to nine local cases of either falciparum or vivax malaria reported) were used. Also, in malaria-free zones in Iran, which had no re-introduction after 1 year since the last reported case, 27 foci with imported malaria cases from abroad were randomly selected and the specificity of the model was determined.

## Results

Table 1 shows 20 explanatory variables that were introduced based on comparing the situation of focus affected by re-introduction with the situation of the same focus 12 months ago, and comparing it with the control focus nearby. Any changes in the 20 selected predicting variables has been considered to be the affecting factors which may cause re-introduction and malaria outbreaks.

**Table 1:** Predictive explanatory variables of malaria re-introduction and/or outbreaks by risk category

Risk assessment variables	Risk classification		
	High	Moderate	Low
Parasite reservoirs			
Population movement of a target focus with endemic areas	Entrance of unpermitted immigrants from neighboring endemic countries to a target focus	Population of a target focus with a history of travelling to neighboring endemic countries	No population movement with endemic malaria areas
Mean score	5	3.99	0.01
Quality of population movement in a target focus	Entrance of unpermitted immigrants from neighboring endemic countries who have no protected settlements that make them exposed to mosquito biting	Population of a target focus with a history of travelling to endemic areas or entrance of unpermitted immigrants from these countries who have protected settlements	No population movement
Mean score	5	3.8	0.2
Proportion of immigrants from neighboring malaria endemic countries in a target focus who were examined for malaria during previous malaria transmission season	Less than 30 %	Between 31 and 60 %	More than 60 %
Mean score	4.7	4	0.3
Target focus classification	New active or residual active	New potential or residual non active	Cleared-up
Mean score	3.9	3.7	1.4
Having a history of malaria outbreaks in a target focus	History of malaria outbreaks during previous 12 months	History of malaria outbreaks during previous 13–36 months	No report of malaria outbreaks during previous 36 months
Mean score	4.1	3.3	1.6
Report of malaria cases (regardless epidemiological classification) in a target focus during previous 3 weeks	Malaria cases were reported every week during previous 3 weeks	Malaria cases were reported within only 1 or 2 weeks during previous 3 weeks	No reported malaria case
Mean score	5.1	2.9	1
Infrastructures, health services and common social behavior in a target focus			
Earliest possible time for a malaria surveillance team to have access to a target focus	The target focus is not accessible by motor vehicles within 7 days	The target focus is accessible between 2 and 7 days by motor vehicles	The target focus is accessible on the first day
Mean score	4.2	3.15	1.65
Earliest possible time for suspected malaria cases in a target focus to have access to malaria diagnosis and treatment services	Malaria diagnosis and treatment services are not accessible within 7 days	Malaria diagnosis and treatment services are accessible between 3 and 7 days	Malaria diagnosis and treatment services are accessible in less than 3 days
Mean score	4	3	2
Sleeping outdoors in a target focus	More than 80 % of people in the target focus have tendency to sleep outdoors	Between 41 and 79 % of people in the target focus have tendency to sleep outdoors	Less than 40 % of people in the target focus have tendency to sleep outdoors
Mean score	4	3.5	1.5
Electricity accessibility	Not accessible	Frequent power outage specially in the evenings and nights	Accessible 24/7
Mean score	4	3.25	1.75
Meteorological variables			
Average daily maximum temperatures in previous 8 weeks	Between 30 and 42 ℃	Between 26 and 30 ℃ or 42 and 44 ℃	Less than 26 ℃ or more than 44 ℃
Mean score	4.9	3.5	0.6
Average daily minimum temperatures in previous 8 weeks	Between 16 and 27.9 ℃	Between 11.5 and 16 ℃ or 27.9 and 29.9 ℃	Less than 11.4 ℃ or more than 30 ℃
Mean score	5.1	3.55	0.35
Average daily relative humidity in the period of previous 4 weeks relative to its previous eight-weeks period	Any increase of more than 22 %	Any increase of more than 17 % and less than 21 %	Any increase of less than 16 % or no increase
Mean score	4.5	3.5	1
Total of rainfall during previous 8 weeks	More than 7 mm	Between 1 and 6 mm	No rainfall
Mean score	5	3	1
Vector variables			
Type of vectors in a target focus^a^	More than two main vector species	At least a main species of vector plus secondary species	Only secondary species
Mean score	4.75	3.25	1
Average time period that common breeding places are existed in a target focus^b^	More than 21 days	Between 8 and 20 days	Between 1 and 7 days
Mean score	4.2	3.25	1.55
Larvae density in a target focus based on randomly larvae collection method	Existence of third and fourth instar larvae and pupae, with majority of first and second instar	Existence of third and fourth instar larvae	No existence of larvae
Mean score	4.38	3.02	1.6
Outdoor resting shelter for malaria vectors in a target focus^c^	A big number of outdoor resting shelters	A few outdoor resting shelters	None
Mean score	5.25	3	0.75
Other variables			
Outbreaks of other diseases in the region during previous 3 months	Yes	No	Unknown
Mean score	4	3.5	1.5
Following malaria transmission season	From March to October	November or February	From December to January
Mean score	4.5	4	0.5

^a^Main vector species in Iran: *Anopheles stephensi*, *culicifacies*, *fluviatilis*. Secondary vector species: *Anopheles d’thali, superpictus, pulcherrimus, sacharovi*

^**b**^ *Breeding places within a radius of 1.6* *km* [35]

^**c**^ *Such as: vast vegetations including trees and shrubs, mountainous areas, mass constructions in residential areas, existence of qanats, wells and water tanks in the majority of residential properties, foci in a radius of 1.5* *km of riverbanks*

**Fig. 2:**
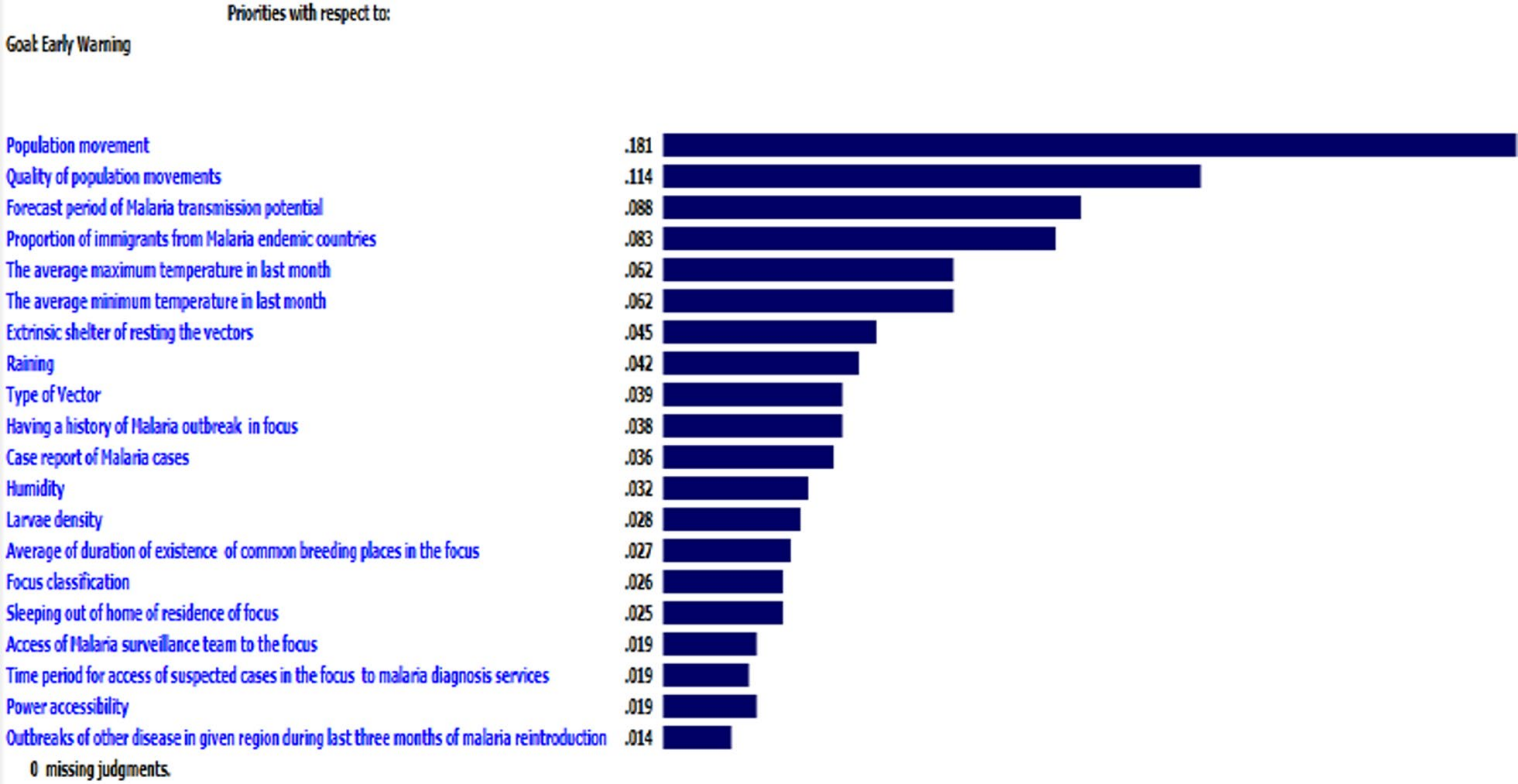
Predictive variables of malaria re-introduction weighted and prioritized by analytical hierarchical process scores

As shown in Fig. 2, based on AHP scores with inconsistency ratio of less than 10 %, ‘population movement of a target focus with endemic areas’ was determined as the pivotal factor (AHP score: 0.181). Moreover, ‘quality of population movement in a target focus’ (AHP score: 0.114), ‘following malaria transmission season’ (AHP score: 0.088), ‘average daily minimum temperature in the previous 8 weeks’ (AHP score: 0.062), ‘outdoor resting shelter for malaria vectors in a target focus’ (AHP score: 0.045), and ‘total of rainfall during the previous 8 weeks’ (AHP score: 0.042), were determined, based on predictive values.

Tables 2, 3, 4, 5 and 6 show multiplicative interaction matrix of parasite reservoir, infrastructures and meteorological and entomological variables by three subcategories of ‘population movement’. In this regard, unauthorized immigrants to Iran from malaria-endemic countries, who had no permanent address and were ambulant, no access of suspected cases in the focus to malaria diagnosis services, the average of daily maximum temperature in the previous 8 weeks, outdoor resting shelter for malaria vectors in a target focus and if the following transmission season is March to October, scored highest for malaria re-introduction and outbreak prediction.

**Table 2:** Multiplicative interaction model of parasite reservoir variables and most deterministic factor of malaria reintroduction by risk classification

Parasite reservoir variables	Population movement of a target focus with endemic areas (Ø)			
	Risk category	High	Moderate	Low
Quality of population movement in a target focus	High	25	19	1
	Moderate	19.5	14.82	0.78
	Low	0.5	0.38	0.02
Proportion of immigrants from neighboring malaria endemic countries in a target focus who were examined for malaria during last malaria transmission season	High	23.5	20	1.5
	Moderate	18.33	15.6	1.17
	Low	0.47	0.4	0.03
Having a history of malaria outbreaks in a target focus	High	20.5	16.5	8
	Moderate	15.99	12.87	6.24
	Low	0.41	0.33	0.16
Report of malaria cases (regardless epidemiological classification) in a target focus during last 3 weeks	High	25.5	14.5	5
	Moderate	19.89	11.31	3.9
	Low	0.51	0.29	0.1
Target focus classification	High	19.5	18.5	7
	Moderate	15.21	14.43	5.46
	Low	0.39	0.37	0.14

**Table 3:** Multiplicative interaction model of community infrastructure variables and most deterministic factor of malaria reintroduction by risk classification

Infrastructures, health services and common social behavior in a given focus	Population movement of a target focus with endemic areas (Ø)			
	Risk category	High	Moderate	Low
Earliest possible time for a malaria surveillance team to have access to a target focus	High	21	15.75	8.25
	Moderate	16.38	12.285	6.435
	Low	0.42	0.315	0.165
Earliest possible time for suspected malaria cases in a target focus to have access to malaria diagnosis and treatment services	High	21	15.75	8.25
	Moderate	16.38	12.285	6.435
	Low	0.42	0.315	0.165
Electricity accessibility	High	20	16.25	8.75
	Moderate	15.6	12.675	6.825
	Low	0.4	0.325	0.175
Sleeping outdoors in a target focus	High	20	17.5	7.5
	Moderate	15.6	13.65	5.85
	Low	0.4	0.35	0.15

**Table 4:** Multiplicative interaction model of meteorological variables and most deterministic factor of malaria reintroduction by risk classification

Meteorological variables (Ɵ)	Population movement of a target focus with endemic areas (Ø)			
	Risk category	High	Moderate	Low
Average daily maximum temperatures in previous 8 weeks	High	24.5	17.5	3
	Moderate	19.11	13.65	2.34
	Low	0.49	0.35	0.06
Average daily minimum temperatures in previous 8 weeks	High	25.5	17.75	1.75
	Moderate	19.89	13.845	1.365
	Low	0.51	0.355	0.035
Total of rainfall during previous 8 weeks	High	25	15	5
	Moderate	19.5	11.7	3.9
	Low	0.5	0.3	0.1
Average daily relative humidity in the period of previous 4 weeks relative to its previous 8 weeks period	High	22.5	17.5	5
	Moderate	17.55	13.65	3.9
	Low	0.45	0.35	0.1

**Table 5:** Multiplicative interaction model of entomological variables and most deterministic factor of malaria reintroduction by risk classification

Vector variables (Ɵ)	Population movement of a target focus with endemic areas (Ø)			
	Risk category	High	Moderate	Low
Average time period that common breeding places are existed in a target focus	High	21	16.25	7.75
	Moderate	16.38	12.675	6.045
	Low	0.42	0.325	0.155
Larvae density in a target focus based on randomly larvae collection method	High	21.9	15.1	8
	Moderate	17.082	11.778	6.24
	Low	0.438	0.302	0.16
Type of vectors in a target focus	High	23.75	16.25	5
	Moderate	18.525	12.675	3.9
	Low	0.475	0.325	0.1
Outdoor resting shelter for malaria vectors in a target focus	High	26.25	15	3.75
	Moderate	20.475	11.7	2.925
	Low	0.525	0.3	0.075

**Table 6:** Multiplicative interaction model of other related variables and most deterministic factor of malaria reintroduction by risk classification

Other variables (Ɵ)	Population movement of a target focus with endemic areas (Ø)			
	Risk category	High	Moderate	Low
Outbreaks of other diseases in the region during previous 3 months	High	20	17.5	7.5
	Moderate	15.6	13.65	5.85
	Low	0.4	0.35	0.15
Following malaria transmission season	High	22.5	20	2.5
	Moderate	17.55	15.6	1.95
	Low	0.45	0.4	0.05

### Calibration of the model

**Fig. 3:**
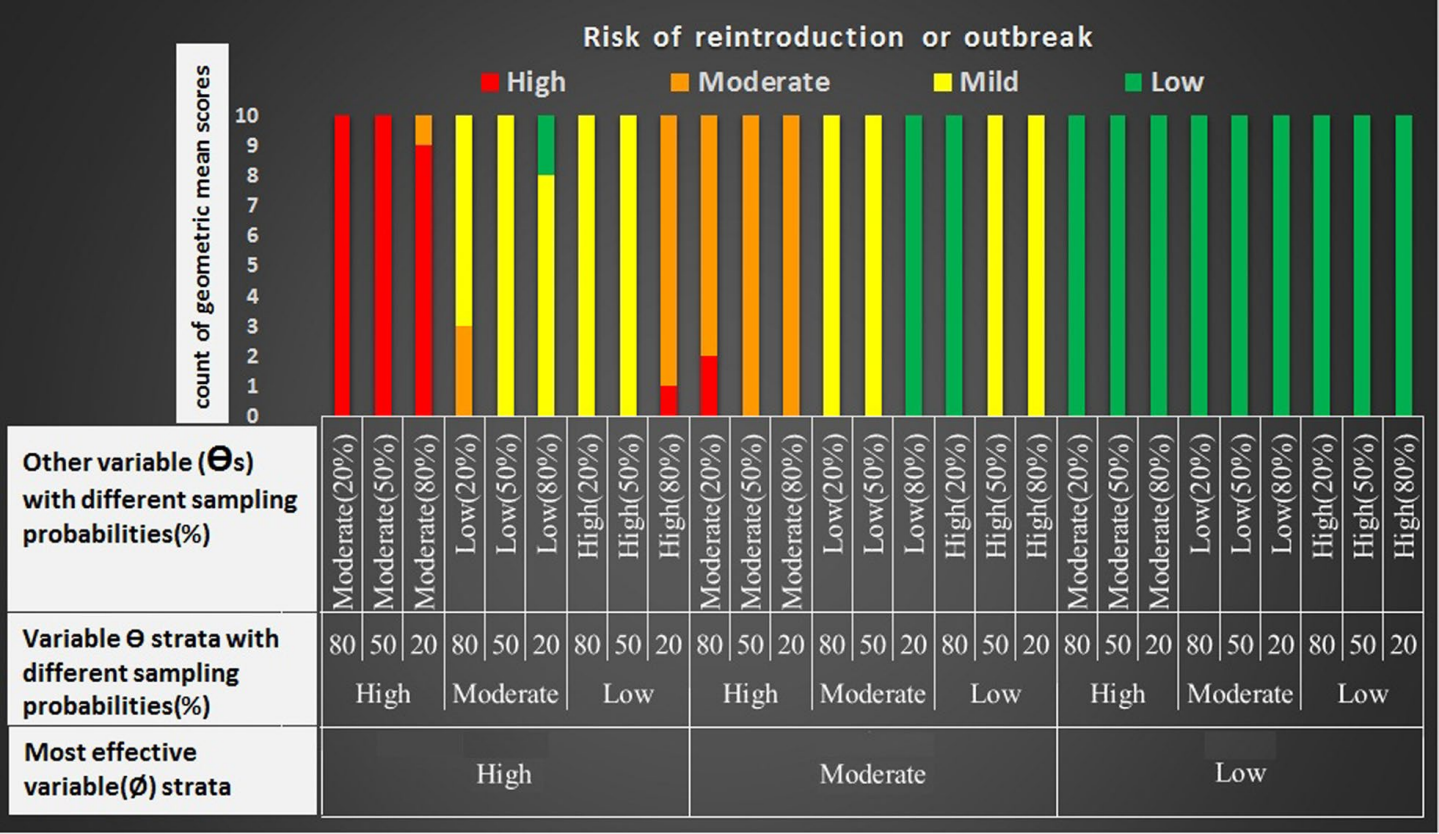
Model calibration tested by resampling different sets of explanatory variables to forecast re-introduction and/or outbreak in a focus

Figure 3 shows the accuracy of the malaria early warning tool improvising various scenarios for explanatory variables with different probabilities. The Figure consists of three main sections based on population movement strata (Ø) including low, moderate and high (bottom row of Figure). The middle and upper rows of the Figure refer to other variables (Ɵi).

### Interpretation of low population movement strata

For a focus with no population movement (as a low-risk stratum), in the presence of different probability sets of other variables, 100 % (90/90 repeated samples) of GMs are in the range of low risk of re-introduction (green).

### Interpretation of moderate population movement strata

In a focus in moderate-risk stratum of population movement, in the presence of other variables with different probabilities, more than 44 % of GMs (40/90) are in the range of mild risk of re-introduction (yellow). In addition, in the presence of other related variable sets in high-risk strata, less than 32 % of GMs (28/90) are in the range of moderate risk of malaria outbreaks (orange). Fewer than 3 % of set samples (2/90) are red (in the case of set samples with 80 % of ƟiH and 20 % of ƟiM), i.e., unadjusted in the range of moderate risk of malaria outbreaks.

### Interpretation of high population movement strata

For high-risk stratum of population movement, in the presence of other related sets of variables with different probabilities, 32 % (29/90) of GMs are in the range of high risk of outbreak (red), and fewer than 8 % (7/90) of GMs are out of range of defined epidemic risk categories. In total, 220 out of 270 tests (81.4 %) were ranked in the range of defined risk categories of re-introduction or malaria outbreaks as proposed by the model (the bars in the same colour).

### Sensitivity and specificity of model

Table 7 shows frequency of risk strata in foci located in different zones of Iran during 2014–2015. Accordingly, the GM scores of risk assessments by the model in foci with a history of re-introduction fall into the ranges of mild risk of re-introduction and moderate risk of re-introduction and the GM scores of risk assessments in foci with a history of outbreak fall into the range of high risk of malaria epidemic (sensitivity = 100 % and positive predictive value of 81.8 %). In addition, the GM scores for control foci fall into the ranges of low risk of malaria re-introduction, and mild risk of malaria re-introduction (specificity = 77 % and negative predictive value of 100 %).

**Table 7:** Risk assessment of foci with a history of reintroduction, outbreak and also malaria free (control) to determine sensitivity, specificity, positive and negative predictive values of the model

Risk category	High	Moderate	Low	Range of GM scores
Foci classification				
Reintroduction	136 (34 %)	149 (37.2 %)	115 (28.8)	9.08–16.35
Outbreak	71 (50.7 %)	56 (40 %)	13 (9.3 %)	17.02–18.96
Control	65 (12.1 %)	80 (14.8 %)	395 (73.1 %)	0.14–5.98

Foci classification	Outbreak	Reintroduction	Control
Test			
+	7	20	6
–	0	0	21
Total	7	20	27
Sensitivity (%)	100 %	100 %	–
Specificity (%)	–	–	77 %
Positive predictive value (%)	81.8 %		–
Negative predictive value (%)	–		100 %

## Discussion

The study highlights predisposing factors of malaria re-introduction and outbreaks in high-risk prone areas in Iran, which is in a pre-elimination setting. The study introduced a new, simple, statistical model to predict malaria re-introduction and outbreaks, with positive predictive value (81.8 %) and negative predictive value (100 %). It incorporates different sets of comprehensive lists of predictive variables in a multiplicative, interactive manner that can be used by decision-makers and end-users at peripheral level to predict malaria re-introduction 8 weeks in advance. Using a tool that can be designed based on a model enables a health system to prioritize allocation of its resources and take necessary action early enough to prevent resurgence of malaria in areas that are already cleared-up but at risk of re-introduction of transmission. Considering this study’s results and proposed methodology, the model can be adjusted based on local circumstances in other countries in order to develop a customized model to meet their requirements. Some of variables that were introduced in the study, including ‘outdoor resting shelter for malaria vectors in a target focus’, ‘quality of population movement in a target focus’, ‘duration average of common breeding places existing in a target focus’, and ‘larvae density in a target focus based on randomly larvae collection method’, were novel predicting factors for malaria outbreaks particulalry in elimiantion setting.

This analysis confirms results from previous studies which indicate that population movement within endemic areas is a key factor for re-introduction of malaria transmission [15, 27]. Based on this study’s findings, population movement, especially with neighbouring endemic countries is the most potential predisposing factor of malaria re-introduction in Iran. While quantifying population movement is a daunting task [4], this study shows that the quality of population movement from endemic areas is of paramount importance, especially when the domicile of a population is missing.

Studies focused on forecasting malaria outbreaks or re-introduction in elimination settings are rare. The results of a study in Spain confirm that seasonality can be an important effective variable in increasing transmission risk. That study showed maximum normalized difference vegetation index (NDVI) values in rice-field areas along with an increase in transmission risk in the period from May to September for *P. falciparum*, and from May to October for *P. vivax*. In addition, it emphasized that an increase in temperature did not mean a malaria transmission risk if accompanied by a precipitation decrease [18]. This favours the study approach to including meteorological variables, such as temperature, precipitation and humidity, in the model. There is enough evidence to support the role of meteorological variables, including rainfall, humidity, maximum and minimum temperatures, in early detection of malaria epidemics through the adaptation of humans, vectors and *Plasmodium* [22, 28–31]. Nevertheless, the advantage of climate data *per se* in malaria incidence prediction is eclipsed by significant uncertainties due to the complexity of ecological indicators, especially in large-scale geographical extents [16]. Other studies stressed the role of environmental variables in malaria transmission and found other variables rather than climatic factors, such as vegetation index, number of malaria cases within the previous month before the prediction, and socio-economic status [32–34].

Given the positive predictive value of the more than 80 % and negative predictive value 100 %, it shows an acceptable level of sensitivity of the model that is a requirement for pre-elimination and elimination settings where the programme should react to every possible active foci. In addition, it will support decision-makers in preventing wasting of resources on foci that are at zero risk of re-introduction.

Following the development of the model to be used in the field, an action plan has been developed to prevent malaria re-introduction/outbreaks. It proposes interventions that should be implemented once the model conveys a risk of re-introduction/outbreaks, e.g., a full service coverage for a focus population, particularly immigrants by early case finding and prompt treatment, enhancement of surveillance team access to a focus, distribution of long-lasting insecticide-treated bed nets to those sleeping outdoors in a target focus, larval source management to eliminate breeding places, as well as adult vector control measures to reduce vectorial capacity of focus.

### Study limitations

Given that the study area is in a pre-elimination setting, a limited number of foci with a history of re-introduction were reported, therefore, the sample size was small. In addition, there are discrepancies between the defined range of temperature and humidity in the model with the theoretical basis of malaria transmission. The reason is that the model was managed to be applicable based on accessible data in the field, i.e., the monthly averages of meteorological data were extracted from ground synoptic weather stations as proximal variables, taking into account the probable systematic errors of the measures. Moreover, the study assumes only a two-dimensional interaction assessment of variables, while it does not take inherent associations of the variables into consideration as well as their probability distributions.

## Conclusion

This study introduced a new, simple, statistical model to forecast malaria re-introduction and outbreak risks in pre-elimination and elimination settings following population movement of the focus with malaria-endemic areas. The model incorporates comprehensive deterministic factors, including 20 variables that are accessible in the field and are easily analysed in an interactive manner. This provides an evidence-based prediction for malaria re-introduction and outbreaks 8 weeks in advance with positive predictive value of 81.8 %, thereby allowing for effective and timely interventions.

## Additional files

10.1186/s12936-016-1192-y An overview of the questioner for gathering information in foci affected by reintroduction of malaria transmission/outbreak.
10.1186/s12936-016-1192-y Product of weights.
10.1186/s12936-016-1192-y Geometric mean calculation.

## Abbreviations

HDI: human development index
AHP: analytic hierarchical process
GM: geometric mean
MEWS: malaria early warning system
ABER: annual blood examination rate
NDVI: normalized difference vegetation index
