# Supplementary material for: Predicting factors for malaria re-introduction: an applied model in an elimination setting to prevent malaria outbreaks
Source: Malar J. 2016 Mar 2;15:138. doi: 10.1186/s12936-016-1192-y (PMC4776358; doi:10.1186/s12936-016-1192-y)
Supplement: Supplementary file 1 — 10.1186/s12936-016-1192-y An overview of the questioner for gathering information in foci affected by reintroduction of malaria transmission/outbreak. [file 12936_2016_1192_MOESM1_ESM.docx]

| An overview of the questioner for gathering information in foci affected by reintroduction of malaria transmission/outbreak | | | | | |
| --- | --- | --- | --- | --- | --- |
| Focus name | Health center name: | District name: | | | |
| Focus population |  |  |  |  |  |
| Time period (Month… to Month) : |  |  |  |  |  |
| District malaria focal point:  Phone number: | Malaria reintroduction onset/detection date:  Day… Month… Year… | Date of completion of the questioner | | | |
| Variables | | | | | Guide |
| Larvae density based on randomly larvae collection method according to the entomological reporting form of the focus in 8th week before malaria reintroduction/outbreak onset | | | | | |
| Existence of 3rd and 4th instar larvae and pupae within the larvae habitats in the area, with majority of 1st and 2nd instar | | | | | Check only one of the three choices!  Please attach the entomological data form. |
| Existence of 3rd and 4th instar larvae within larvae habitats in the area | | | | |  |
| The larvae are non-existent within larvae habitats in the area (inactive larvae habitats) | | | | |  |
| Duration average  of common breeding places existing in a target focus in 8th week before malaria reintroduction/outbreak onset | | | | | |
| Life span more than 21 days. | | | | | The answer to this question can be drawn from the district entomological data.Check only one of the three choices! Also please attach the entomological data form. |
| Life span Between 8 and 20 days | | | | |  |
| Life span  Between 1 and 7 days | | | | |  |
| Existence of main vectors (*Anopheles stephensi*, *culicifacies*, *fluviatilis*) in the focus in 8th week before malaria reintroduction/outbreak onset  . | | | | | |
| More than 2 main vector species | | | | | The answer to this question can be drawn from the district entomological data Check only one of the four choices! Also please attach the entomological data form. |
| At least a main species of vector plus secondary species | | | | |  |
| Only secondary species | | | | |  |
| Malaria cases report of the focus within the last 12 weeks before malaria reintroduction/outbreak onset | | | | | |
| Malaria cases were reported every week during last three weeks | | | | | .  The answer to this question can be drawn from the district outbreak reports.  Check only one of the four choices! |
| Malaria cases were reported within only one or two weeks  during last three weeks | | | | |  |
| No reported malaria case | | | | |  |
| The number of malaria cases in the focus within the last 12 weeks before malaria reintroduction/outbreak onset  . | | | | | |
| There was an increase, and the majority were passive cases. | | | | | The answer to this question can be drawn from the district outbreak reports Check only one of the four choices! |
| There was an increase, and the majority were active cases. | | | | |  |
| The number of cases were as expected. | | | | |  |
| No malaria cases were reported. | | | | |  |
| A history of indoor residual spraying operations in the focus within the last three months before the epidemic onset | | | | Yes | The answer to this question can be drawn from the district indoor residual spraying operations reports. |
|  |  |  |  | No |  |
|  | | | | | |
| A history of malaria outbreaks in the focus within the last three years | | | | | |
| Within the period of less than 12 months, were any malaria outbreak reported? | | | | | The answer to this question can be drawn from the district outbreak report.  Check only one of the three choices! |
| Between the last 12-36 month-period, were any malaria outbreak reported? | | | | |  |
| Within the last 36 months, no malaria outbreak were reported. | | | | |  |
| Population movement of target focus before outbreak onset within the previous three months | | | | | |
| Entrance of unpermitted immigrants from neighboring endemic countries to a target focus | | | | | .  The answer to this question can be drawn from health houses or health centers reports. Check only one of the three choices. Among 2 and 3 select 2 |
| Population of a target focus with a history of travelling to neighboring endemic countries | | | | |  |
| No population movement with endemic malaria areas | | | | |  |
| **Earliest possible time for a malaria surveillance team to have access to a target focus** | | | | | |
| The target focus is not accessible by motor vehicles within seven days | | | | | The answer to this question can be drawn from the information of the network development office. Check only one of the three choices! |
| The target focus is accessible between 2 and 7 days by motor vehicles | | | | |  |
| The target focus is accessible on the first day | | | | |  |
| Earliest possible time for suspected malaria cases in a target focus to have access to malaria diagnosis and treatment services | | | | | |
| Malaria diagnosis and treatment services are not accessible within seven days | | | | | The answer to this question can be drawn from health houses or health centers reports.  Check only one of the three choices! |
| Malaria diagnosis and treatment services are accessible between 3 and 7 days | | | | |  |
| Malaria diagnosis and treatment services are accessible in less than 3 days | | | | |  |
| Concurrent outbreak of other diseases within the previous eight weeks before malaria reintroduction/outbreak onset | | | Yes | | ا  The answer to this question can be drawn from health houses or health centers reports. |
|  |  |  | NO | |  |
|  |  |  | Unknown | |  |
|  | | | | | |
| Access to electricity in the focus within the previous eight weeks before malaria reintroduction/outbreak onset | | | | | |
| Not accessible | | | | | The answer to this question can be drawn from the existing information in the focus.  Check only one of the three choices! |
| Frequent power outage specially in the evenings and nights | | | | |  |
| Accessible 24/7 | | | | |  |
| Existence of outdoor resting shelters within the previous eight before malaria reintroduction/outbreak onset | | | | | |
| A big number of outdoor resting shelters | | |  | | The answer to this question can be drawn from health houses or health centers information. Check only one of the three choices! |
| A few outdoor resting shelters | | |  | |  |
| None | | |  | |  |
| Quality of population movement in a target focus | | | | | |
| Entrance of unpermitted immigrants from neighboring endemic countries who have no protected settlements that make them exposed to mosquito biting | | | | | Check only one of the three choices! |
| Population of a target focus with a history of travelling to endemic areas or entrance of unpermitted immigrants from these countries who have protected settlements | | | | |  |
| No population movement | | | | |  |
| Target focus classification | | | | | |
| New active or Residual active | | | | | Check only one of the three choices! |
| New potential or Residual non active | | | | |  |
| Cleared-up | | | | |  |
| Proportion of immigrants from neighboring malaria endemic countries in a target focus who were examined for malaria during last malaria transmission season | | | | | |
| Less than 30% | | | | | Check only one of the three choices!  Reference malaria labs data sheet |
| Between 31-60% | | | | |  |
| More than 60% | | | | |  |
| The proportion of the persons sleeping outdoors without bed nets within the previous eight weeks before malaria reintroduction/outbreak onset | | | | | |
| More than 80% of people in the target focus have tendency to sleep outdoors | | | | | The answer to this question can be drawn from households survey results  Check only one of the four choices! |
| Between 41-79% of people in the target focus have tendency to sleep outdoors | | | | |  |
| Less than 40% of people in the target focus have tendency to sleep outdoors | | | | |  |
| **Average daily maximum temperatures in the previous eight weeks** | | | | |  |
| **Average daily minimum temperatures in the previous eight weeks** | | | | |  |
| **Average daily relative humidity in the period of previous four weeks** | | | | |  |
| **Average daily relative humidity in period of 4-8 week period** | | | | |  |
| **Total of rainfall during previous eight weeks** | | | | |  |
| **Total rainy days during previous eight weeks** | | | | |  |

| The number of cases with fever approaching the health facilities within the last three months before outbreak onset | | | | | | | | | | | | | |
| --- | --- | --- | --- | --- | --- | --- | --- | --- | --- | --- | --- | --- | --- |
|  | The week reintroduction occurred | | | The previous week before the reintroduction | | The previous two weeks before the reintroduction | | The previous three weeks before the reintroduction | | The previous four weeks before the reintroduction | | The previous five weeks before the reintroduction | |
|  | Passive | Active | | Passive | Active | Passive | Active | Passive | Active | Passive | Active | Passive | Active |
| Number of patients |  |  | |  |  |  |  |  |  |  |  |  |  |
|  | The previous six weeks before the reintroduction | | | The previous seven week before the reintroduction | | The previous eight weeks before the reintroduction | | The previous nine weeks before the reintroduction | | The previous ten weeks before the reintroduction | | The previous eleven weeks before the reintroduction | |
|  | Passive | Active | | Passive | Active | Passive | Active | Passive | Active | Passive | Active | Passive | Active |
| Number of patients |  | |  |  |  |  |  |  |  |  |  |  |  |

| The number of malaria cases (confirmed with blood smears or RDT kits) within the last three months before outbreak onset | | | | | | | | | | | | | |
| --- | --- | --- | --- | --- | --- | --- | --- | --- | --- | --- | --- | --- | --- |
|  | The week reintroduction occurred | | | The previous week before the reintroduction | | The previous two weeks before the reintroduction | | The previous three weeks before the reintroduction | | The previous four weeks before the reintroduction | | The previous five weeks before the reintroduction | |
|  | Passive | Active | | Passive | Active | Passive | Active | Passive | Active | Passive | Active | Passive | Active |
| The number of malaria cases |  |  | |  |  |  |  |  |  |  |  |  |  |
|  | The previous six weeks before the reintroduction | | | The previous seven week before the reintroduction | | The previous eight weeks before the reintroduction | | The previous nine weeks before the reintroduction | | The previous ten weeks before the reintroduction | | The previous eleven weeks before the reintroduction | |
|  | Passive | Active | | Passive | Active | Passive | Active | Passive | Active | Passive | Active | Passive | Active |
| The number of malaria cases |  | |  |  |  |  |  |  |  |  |  |  |  |
|  |  | |  |  |  |  |  |  |  |  |  |  |  |
