# Supplementary material for: Predicting factors for malaria re-introduction: an applied model in an elimination setting to prevent malaria outbreaks
Source: Malar J. 2016 Mar 2;15:138. doi: 10.1186/s12936-016-1192-y (PMC4776358; doi:10.1186/s12936-016-1192-y)
Supplement: Supplementary file 2 — 10.1186/s12936-016-1192-y Product of weights. [file 12936_2016_1192_MOESM2_ESM.docx]

Additional file 2: Product of weights

Suppose $\varphi$indicate the most effective variable diagnosed by AHP method and all other variables are shown as $\theta_{i};i=1,...,19$. Sub-scripts of H, M, and L represent high, moderate, and low levels of variables, respectively. Take $\varphi_{j}$ and $\theta_{ij}$ ($j=H,M,L$) as corresponding weight of levels of variables determined by the difference of relative frequency of each variable in cases and controls. Considering three levels for each variable, Table A1 represents the product of weights for combination of three levels of the most effective variable with each of the 19 remaining variables.

**Table A1 Combination of three levels of the most effective variable with each of the 19 remaining variables to predict malaria re-introduction and outbreaks in the next eight weeks**

| Each of other 19 variables | | The most effective variable by AHP method (Ø) | | |
| --- | --- | --- | --- | --- |
|  |  | High | Moderate | Low |
| $\begin{matrix} \theta_{i} \\ i=1,...,19 \end{matrix}$ | High | $\varphi_{H}\times\theta_{iH}$ | $\varphi_{M}\times\theta_{iH}$ | $\varphi_{L}\times\theta_{iH}$ |
|  | Moderate |  | $\varphi_{M}\times\theta_{iM}$ | $\varphi_{L}\times\theta_{iM}$ |
|  | Low | $\varphi_{H}\times\theta_{iL}$ | $\varphi_{M}\times\theta_{iL}$ | $\varphi_{L}\times\theta_{iL}$ |

For example:

|  | | Population movement of a target focus with endemic areas | | |
| --- | --- | --- | --- | --- |
|  |  | High | Moderate | Low |
| Quality of population movement | High | 25 | 19.50 | 0.50 |
|  | Moderate | 19 | 14.82 | 0.38 |
|  | Low | 1 | 0.78 | 0.02 |
