# Supplementary material for: Predicting factors for malaria re-introduction: an applied model in an elimination setting to prevent malaria outbreaks
Source: Malar J. 2016 Mar 2;15:138. doi: 10.1186/s12936-016-1192-y (PMC4776358; doi:10.1186/s12936-016-1192-y)
Supplement: Supplementary file 3 — 10.1186/s12936-016-1192-y Geometric mean calculation. [file 12936_2016_1192_MOESM3_ESM.docx]

Additional file 3: **Geometric mean calculation**

According to Appendix I, there are 19 tables with nine cells. Table A2 represents the geometric mean of values in corresponding cells of those Tables.

**Table A2 Geometric mean calculation to predict malaria re-introduction and outbreaks for the next eight weeks**

| Risk strata | Ø High | Ø Moderate | Ø Low |
| --- | --- | --- | --- |
| Ɵ High |  |  |  |
| Ɵ Moderate |  |  |  |
| Ɵ Low |  |  |  |

: indicates the product of elements over the 19 remaining variables except the most effective variable.

For instance, according to 19 matrix outputs in Appendix 1, the geometric means (GMs) in corresponding cells were obtained as follow:

| Risk strata | Ø High | Ø Moderate | Ø Low |
| --- | --- | --- | --- |
| Ɵ High | 22.35 | 16.81 | 4.72 |
| Ɵ Moderate | 17.48 | 13.11 | 3.69 |
| Ɵ Low | 0.45 | 0.34 | 0.09 |
